# Clinicopathologic Characteristics and Clinical Outcomes of Patients with Testicular Mesothelioma

**DOI:** 10.1245/s10434-025-17978-3

**Published:** 2025-07-30

**Authors:** Prashasti Agrawal, Fady Baky, Judy Sarungbam, Joel Sheinfeld, Victor E. Reuter, Andrea Cercek, Garrett M. Nash, Jennifer L. Sauter, William D. Travis, Charles B. Simone, George J. Bosl, Marjorie G. Zauderer, Richard S. Matulewicz, Michael Offin

**Affiliations:** 1 Department of Medicine, Memorial Sloan Kettering Cancer Center, New York, NY 10065 USA; 2 Department of Surgery, Memorial Sloan Kettering Cancer Center, New York, USA; 3 Department of Pathology, Memorial Sloan Kettering Cancer Center, New York, USA; 4 Department of Radiation Oncology, Memorial Sloan Kettering Cancer Center and New York Proton Center, New York, USA; 5 Department of Hematology and Medical Oncology, Westchester Medical Center, New York, USA

**Keywords:** Testicular mesothelioma, Orchiectomy, Rare tumor, Epithelioid, Non-epithelioid

## Abstract

**Background:**

Mesothelioma of the tunica vaginalis testes (testicular mesothelioma [TM]) is a rare tumor, comprising less than 5% of mesotheliomas. Surgical intervention is the current standard of care, whereas the role of systemic therapies remains undefined.

**Methods:**

We retrospectively reviewed 36 patients with pathologically confirmed TM treated at Memorial Sloan Kettering Cancer Center (MSK) between January 1996 to May 2023. Clinicopathologic data, treatments received, and clinical outcomes were reported. Surgical pathology samples, when available, underwent next-generation sequencing (NGS) using MSK-IMPACT. Overall survival (OS) was calculated by using the Kaplan–Meier method.

**Results:**

Median age at diagnosis was 64 years (range 24–93). Histologically, 22 (61%) tumors were epithelioid, ten (28%) were biphasic, three (8%) were sarcomatoid, and one (3%) was unable to be further classified. Ten patients (28%) had metastatic disease at initial diagnosis, and 13 more (36%) eventually developed metastatic disease. Orchiectomy was performed in 34 (94%) patients and retroperitoneal lymph node dissection (RPLND) in 12 (33%); 15 (42%) received systemic therapy and five (14%) radiation therapy. Ten samples underwent somatic NGS: five (50%) had *CDKN2A/B*, four (40%) had *NF2*, and two (20%) had *BAP1* alterations. At a median follow up of 3.5 years, median OS was 4.5 years.

**Conclusions:**

This is the largest single-institution series and genomic dataset for TM. We report unique clinicopathologic and genomic features that distinguish TM from other mesotheliomas, including a lower frequency of *BAP1* alterations. Owing to the rarity of this disease, further refinement of the sequence and necessity of surgical and systemic treatment is necessary.

**Supplementary Information:**

The online version contains supplementary material available at 10.1245/s10434-025-17978-3.

Mesothelioma of the tunica vaginalis testis (also known as testicular mesotheliomas [TM]) is a rare, understudied tumor, representing less than 5% of all mesotheliomas. Mesotheliomas arising from different sites of origin (pleural, peritoneal, pericardial) have unique clinicopathologic features and represent distinct disease entities.^1–3^ Testicular mesotheliomas also may have distinct clinicopathologic features and outcomes; however, few studies are available on this topic, and the literature primarily consists of case reports or small case series.^4,5^ Patients with TM often present with nonspecific symptoms such as scrotal swelling, a palpable scrotal mass, or hydrocele.^6–8^ Thus, preoperative diagnosis of TM remains challenging owing to its nonspecific clinical presentation and radiographic findings, and most cases are diagnosed intraoperatively or postoperatively.^9^ A high level of clinical suspicion and expert pathologic review are needed to obtain an accurate diagnosis. The main treatment modality is surgical (orchiectomy) with or without wide local excision of the soft tissue, including potential scrotectomy, but there are no established guidelines for performing additional surgical resection, including potentially pelvic lymphadenectomy (PLND), inguinal lymphadenectomy (ILND), and/or retroperitoneal lymph node dissection (RPLND).^10^ In the absence of definitive data regarding systemic therapies and owing to a poor understanding of the varying metastatic potential following removal of the primary tumor, aggressive surgery is often utilized. The roles of systemic therapies and radiotherapy remain undefined and are extrapolated from the more common diffuse pleural mesothelioma (DPM).^10^ Prognosis for TM is often poor, with rates of distant metastases reported as high as 35% and 5-year survival rates less than 50%.^5,11^

Additionally, the genomic landscape of TM is not defined. Disease-specific research is essential to improve the understanding of the pathologic characteristics, tumor molecular alterations, and natural histories of patients with TM to develop evidence-based treatment guidelines, define the role of systemic treatments and surgery in the management of these patients, and improve patient outcomes. This report presents the largest single-institutional series and genomic dataset of patients with TM with detailed clinical annotations that describe the clinicopathologic characteristics, molecular features, treatment histories, clinical outcomes, and genomic comparisons with other mesotheliomas.

## Methods

Patients with pathologically confirmed TM at Memorial Sloan Kettering Cancer Center (MSK) between January 1996 and May 2023 were identified and reviewed and followed through May 2024. The study was approved by the MSK institutional review board and conducted in accordance with the US Common Rule. All patients with available archival material had their tissue re-reviewed to confirm diagnosis and histologic subtype by dedicated genitourinary pathologists and discussed with the multidisciplinary MSK Mesothelioma Program. Pathologic assessments, including immunohistochemistry, were performed in accordance with clinically validated protocols with appropriate internal controls.

Identified patients had their baseline demographics, clinicopathologic features, treatment histories, and patient outcomes collected. Overall survival (OS) was assessed from the date of initial pathologic diagnosis until the date of death or last follow-up. Recurrence-free survival (RFS) was assessed in patients with nonmetastatic disease at diagnosis from the date of radical orchiectomy until the date of disease recurrence or last follow-up. Between-group comparisons were made by using the log-rank test with *p*-value < 0.05 considered statistically significant. Survival analyses were performed using the Kaplan–Meier method in GraphPad Prism version 10 (San Diego, CA). Somatic next-generation sequencing (NGS) and germline testing were performed using the MSK-IMPACT platform for all patients who provided appropriate consent and for whom tumor tissue/matched normal blood was available.^12^ Somatic alterations were annotated along with assessment of tumor mutation burden (TMB; mutations/megabase [mut/Mb]) and fraction of genome altered (FGA). Germline assessments were performed on all patients who gave prospective informed consent. To compare the genomic features of TM with other mesotheliomas, we utilized cohorts of patients with malignant peritoneal mesothelioma (MPeM, *n* = 50) from MSK and DPM from The Cancer Genome Atlas (*n* = 74) to compare the prevalence of identified alterations using the chi-square test in GraphPad Prism version 10 (San Diego, CA).^1,13^

## Results

### Patient Characteristics

A total of 36 patients were identified following clinical and pathologic confirmation of TM with a median age at diagnosis of 64 years (range, 24–93 years; Table 1). Twenty-two (61%) patients had epithelioid tumors, ten (28%) had biphasic tumors, three (8%) had sarcomatoid tumors, and one (3%) had a tumor with unclassified histology due to lack of archival tissue for review (Supplemental Fig. 1). At diagnosis, four (11%) patients had involvement of the rete testis, nine (25%) patients of the epididymis, and 15 (42%) patients of the spermatic cord. Ten (28%) patients had metastatic disease at diagnosis, most commonly involving the inguinal and retroperitoneal lymph nodes (6 patients, 17%) and lung parenchyma (3 patients, 8%). During their disease course, an additional 13 (36%) patients developed metastatic disease, most commonly involving the inguinal and retroperitoneal lymph nodes (12 patients, 33%) and lung parenchyma (3 patients, 8%). Eight of 36 (22%) patients self-reported potential classical occupational exposure to asbestos and 18 of 36 (50%) patients reported a history of tobacco use with a median of 20 pack-years (range 2–90 pack-years).

**Table 1:** Clinicopathologic characteristics of patients with testicular mesothelioma (*N* = 36)

Median age at diagnosis (range), years	64 (24–93)
*Race*	
White, *n* (%)	31 (86%)
Non-White, *n* (%)	5 (14%)
*Ethnicity*	
Hispanic	3 (8%)
Non-Hispanic	21 (58%)
Not specified	12 (33%)
*Histology*	
Epithelioid, *n* (%)	22 (61%)
Biphasic, *n* (%)	10 (28%)
Sarcomatoid, *n* (%)	3 (8%)
Not otherwise specified, *n* (%)	1 (3%)
*Testicular involvement at diagnosis*	
Rete testis, *n* (%)	4 (11%)
Epididymis, *n* (%)	9 (25%)
Spermatic cord, *n* (%)	15 (42%)
*Metastatic disease at diagnosis, n (%)*	10 (28%)
Inguinal/retroperitoneal lymph nodes, *n* (%)	6 (17%)
Lung parenchyma, *n* (%)	3 (8%)
Other^a^, *n* (%)	5 (14%)
*Metastatic disease ever, n (%)*	23 (64%)
Inguinal/retroperitoneal lymph nodes, *n* (%)	18 (50%)
Lung parenchyma, *n* (%)	6 (17%)
Other^a^, *n* (%)	11 (31%)
*Self-reported classical occupational asbestos exposure*	
Yes, *n* (%)	8 (22%)
No/Unknown, *n* (%)	28 (78%)
*History of tobacco use*	
Ever, *n* (%)	18 (50%)
Never, *n* (%)	18 (50%)

^a^Other sites of metastatic disease include the peritoneum, pleura, bone, thoracic lymph nodes, pericardial lymph nodes, liver, and skin

**Fig. 1:**
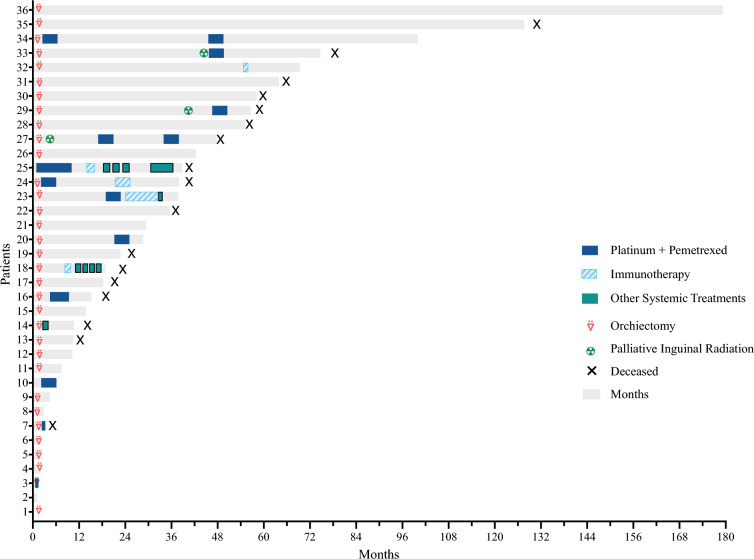
Swimmers plot annotating the treatment modalities, duration on therapy, and survival for patients with testicular mesothelioma (*n* = 36) from date of pathologic diagnosis. Patients not marked with an X were alive at the time of last follow-up

### Treatments

Thirty-four (94%) patients underwent surgical resection with orchiectomy (Table 2); 15 (42%) underwent initial planned inguinal radical orchiectomy, 13 (36%) underwent initial hydrocelectomy followed by subsequent inguinal radical orchiectomy, four (11%) underwent initial inguinal hernia repair with intraoperative orchiectomy, and two (6%) underwent initial scrotal exploration or hydrocelectomy with subsequent conversion to scrotal orchiectomy. Two patients underwent initial hydrocelectomy and were found to have metastatic disease and did not undergo subsequent radical orchiectomy, and 16 (44%) patients also underwent hemiscrotectomy.

**Table 2:** Treatments received by patients with testicular mesothelioma (*N* = 36)

Surgical treatment	
Radical orchiectomy	34 (94%)
*Initial surgery, n (%)*	
Inguinal radical orchiectomy	15 (42%)
Hydrocelectomy	13 (36%)
Inguinal hernia repair	4 (11%)
Scrotal exploration	2 (6%)
*Subsequent surgeries, n (%)*	
Hemiscrotectomy	16 (44%)
Retroperitoneal lymph node dissection	12 (33%)
Pelvic lymph node dissection	9 (25%)
Inguinal lymph node dissection	8 (22%)
*Retroperitoneal lymph node dissection, n (%)*	12 (33%)
Bilateral	9 (25%)
Unilateral	2 (6%)
Unknown	1 (3%)
Median lymph node yield (IQR)	40 [23.57]
*Systemic treatments*	
Total, *n* (%)	15 (42%)
*Number of lines received, n (%)*	
0	21 (58%)
1	9 (25%)
2	2 (6%)
≥ 3	4 (11%)
*Platinum-pemetrexed chemotherapy, n (%)*	
First-line	12^a^ (33%)
Immune checkpoint inhibitors, *n* (%)	5 (14%)
First-line	2 (6%)
Second-line	3 (8%)
Other treatments^b^, *n* (%)	7 (19%)
*Radiation therapy*	
Inguinal, *n* (%)	3 (8%)
Distant, *n* (%)	2 (6%)

^a^Two patients were rechallenged with platinum/pemetrexed in later lines of therapy

^b^These patients received one or more of the following treatments: carboplatin/paclitaxel, bevacizumab, gemcitabine, vinorelbine, and clinical trial drugs including tyrosine kinase inhibitors and drugs targeting PI3K, NEDD8-activating enzyme, mesothelin, and MAT2A

Lymph node dissections were performed in 17 patients (47%). Twelve (33%) patients underwent RPLND—nine bilateral and two unilateral. One patient underwent RPLND prior to referral to our center and the limits of his dissection are unknown. Four patients had imaging demonstrative of retroperitoneal lymphadenopathy prior to RPLND, among whom three were found to have disease in the retroperitoneum. Eight patients underwent RPLND without evidence of advanced disease, among whom three were found to have disease in the retroperitoneum. Nine (25%) patients had PLND, one of whom was found to have positive pelvic nodes. Eight (22%) patients had ILND, six of whom were found to have positive inguinal nodes.

Fifteen (42%) patients received disease-specific systemic treatment during the follow-up period. One patient received adjuvant treatment with four cycles of cisplatin/pemetrexed following surgery, whereas the remaining 14 patients received systemic treatment for metastatic or recurrent disease. Among patients who received systemic therapy, the median number of treatment lines received was one (range, 1–6). Nine (25%) patients received one line of systemic therapy, two (6%) patients received two lines of systemic therapy, and one (3%) patient each received three, four, five, and six lines of systemic therapy. Treatments included platinum-pemetrexed chemotherapy in the first line (12 patients, 33%), immune checkpoint inhibitors in the first line (2 patients, 6%) or second line (3 patients, 8%), and other types of chemotherapies and clinical trial targeted treatments (7 patients, 19%; Fig. 1). Radiation therapy was used in five (14%) patients: three (8%) received inguinal irradiation, and two (6%) received palliative irradiation to sites of distant metastases.

### Survival Analyses

**Fig. 2:**
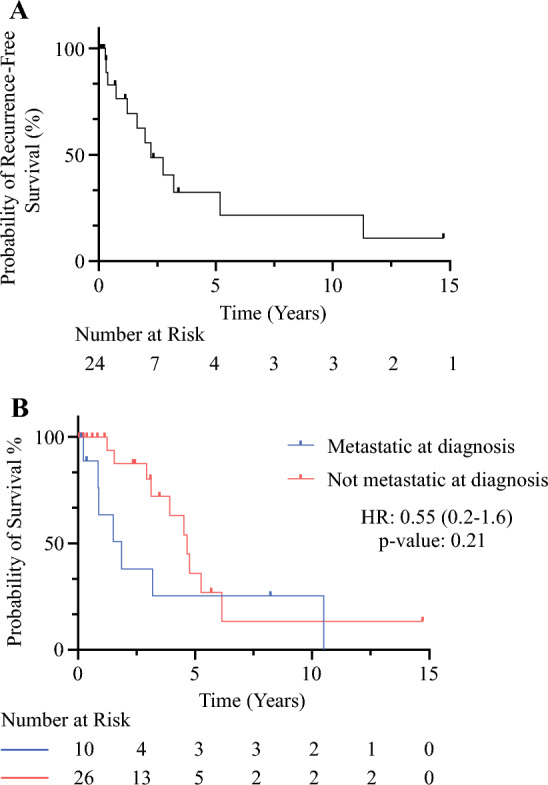
**A** Probability of recurrence-free survival in patients with non-metastatic testicular mesothelioma at diagnosis from the date of radical orchiectomy (*n* = 24). Number of patients at risk at various time points are shown below in the graph. Two patients with non-metastatic disease at diagnosis were not included in this analysis because one underwent orchiectomy at another institution and one had documentation of disease recurrence on outside follow-up but dates could not be ascertained for either. **B** Probability of survival in patients with metastatic (*n* = 10) vs. nonmetastatic (*n* = 26) testicular mesothelioma from the date of pathologic diagnosis. Median overall survival between patients with metastatic and non-metastatic disease at diagnosis did not differ (log-rank test, *p* = 0.21). Numbers of patients at risk at various time points are shown below the graph. Hazard ratio (HR) ± 95% confidence interval is shown

Among patients with nonmetastatic disease at diagnosis who underwent radical orchiectomy, median RFS was 2.2 (range 0.1–14.7) years with a median follow-up time of 3.4 years (Fig. 2A).

Across all patients, median OS was 4.5 (range 0.1–14.7) years with a median follow-up time of 3.5 years. Among the ten patients with metastatic disease at diagnosis, median OS was 1.9 (range 0.1–10.5) years, and median follow-up time was 8.2 years (Fig. 2B). For the 26 patients with nonmetastatic disease at diagnosis, the median OS was 4.7 (range 0.1–14.7) years, and median follow-up time was 3.1 years. The median OS did not significantly differ between patients with metastatic and nonmetastatic disease at diagnosis (log-rank test, *p* = 0.21).

Among the 22 patients with epithelioid histology, median OS was 4.7 years, and median follow-up time was 3.1 years. For the 13 patients with nonepithelioid histology, median OS was 3.9 years, and median follow-up time was 3.5 years. The median OS did not significantly differ between patients with epithelioid and nonepithelioid histology (log-rank test, *p* = 0.33).

### Genomics

**Fig. 3:**
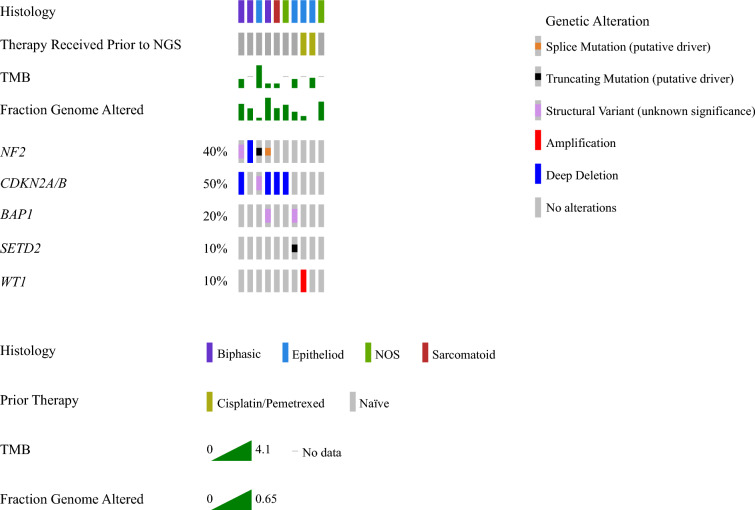
Oncoprint of tumor samples from patients with testicular mesothelioma evaluated with next-generation sequencing (NGS) on the MSK-IMPACT platform (*n* = 10). The percentage of patients with each genetic alteration is indicated to the left of the row. Also shown is the histologic subtype, tumor mutational burden (TMB, mutations/Mb), fraction of genome altered, and whether cisplatin/pemetrexed therapy was received prior to NGS. Each column represents an individual patient’s tumor sample

Next-generation sequencing using MSK-IMPACT was performed on all patients with available tumor tissue, matched normal blood, and who underwent informed consent (n = 10; Fig. 3). Median TMB was 0.85 (range, 0–4.1) mut/Mb. Genetic co-alterations were most common in *NF2* (*n* = 4, 40%), *CDKN2A* (*n* = 4, 40%), *CDKN2B* (*n* = 5, 50%), and *BAP1* (*n* = 2, 20%). There was a higher frequency of *CDKN2A/B* alterations in the TM cohort compared with our previously published cohort with MPeM who underwent NGS at MSK (8%, *p* = 0.0007; Table 3).^1^ Similarly, there was a lower frequency of *BAP1* alterations in our cohort compared with our previously published cohort of MPeM (60%, *p* = 0.02) and a publicly available cohort of DPM from The Cancer Genome Atlas (55%, *p* = 0.04).^1,13^

**Table 3:** Comparison of genomic alterations in patients with testicular mesothelioma (*n* = 10), malignant peritoneal mesothelioma (*n* = 50), or diffuse pleural mesothelioma datasets from The Cancer Genome Atlas (*n* = 74).

Alteration	TM, % (*n* = 10)	MPeM, % (*n* = 50)	DPM, % (*n* = 74)	TM vs. MPeM *p* value	TM vs. DPM *p* value	MPeM vs. DPM *p* value
*NF2*	40	24	34	0.3	0.7	0.24
*CDKN2A/B*	50	8	42	**0.0007**	0.63	**< 0.0001**
*BAP1*	20	60	55	**0.02**	**0.04**	0.61
*TP53*	0	16	14	0.17	0.22	0.7
*SETD2*	10	22	18	0.39	0.55	0.54

Alterations were detected using next-generation sequencing on the MSK-IMPACT platform from the publicly available The Cancer Genome Atlas

All *p*-values were calculated by using chi-squared test. Bold denotes *p* < 0.05. *DPM* diffuse pleural mesothelioma; *MPeM* malignant peritoneal mesothelioma; *TM* testicular mesothelioma

Germline genetic testing using MSK-IMPACT was performed on six patients who provided separate prospective informed consent (Table 4). Among these, one patient (17%) had a pathogenic germline alteration in *NTHL1*. No other patients had adequate material available and/or gave consent prior to death for genomic testing.

**Table 4:** Germline alterations in patients with testicular mesothelioma (*n* = 6) with next-generation sequencing determined using the MSK-IMPACT platform

Age at diagnosis (years)	Germline mutation	Tumor histology	Cancers in first-degree relatives
68	*NTHL1* Q90	Epithelioid	Lung cancer; possible colon or prostate cancer
73	None detected	Epithelioid	Lymphoma, bladder cancer, small cell lung cancer
31	None detected	Epithelioid	Prostate cancer
44	None detected	Epithelioid	None
62	None detected	Sarcomatoid	None
71	None detected	Epithelioid	Hepatocellular carcinoma; head and neck cancer

## Discussion

Testicular mesothelioma is a rare malignancy for which disease-specific research is lacking. In this series, we describe the clinicopathologic and genomic characteristics, treatment histories, and survival outcomes of 36 patients with TM seen at a large academic cancer center. We report unique features of this disease that suggest TM may be a distinct entity from other mesotheliomas and highlight the need to establish disease-specific treatment guidelines beyond orchiectomy.

In this cohort, the median age at diagnosis (64 years) was younger compared with patients with DPM where the median age at diagnosis is 70 years and was similar compared with patients with peritoneal mesothelioma where the median age at diagnosis is 63 years.^14,15^ We found that just greater than 60% of patients had epithelioid tumors, a proportion similar to that reported in other TM studies and in DPM.^5,16^ Understanding the histologic breakdown of TM is important, because histology in other mesotheliomas is prognostic for survival and predictive of systemic therapy response in the first-line setting.^17,18^ Specifically, in DPM, nonepithelioid histology is correlated with more aggressive features and worse prognosis. In our cohort of patients with TM, we did not find a statistically significant difference in OS between patients who have epithelioid versus nonepithelioid histology. Larger cohorts of patients with TM are needed to study the prognostic significance of histology and potential association with OS. We observed a higher median OS for patients with nonmetastatic disease at diagnosis (4.7 years) compared with those with metastatic disease at diagnosis (1.9 years), although this result was not statistically significant, likely owing to the small sample size.

Our findings generally align with a prior SEER database series of 113 TM patients reported by Nazemi et al.^5^, which also showed a median age of 64 years, predominantly epithelioid histology followed by biphasic histology, similar rates of nodal involvement, and high rates of surgical resection. Rates of metastatic disease at diagnosis were higher in our cohort; however, in the SEER series, disease stage and extent were not available in almost a third of patients, and the cohort included patients who received care in both community settings and tertiary care centers. Overall survival in our cohort (4.5 years) was similar to the SEER series but higher than other smaller studies, which have reported a median OS of around 2 years.^6,10,19^ Nazemi et al. found that the biphasic subtype was associated with worse survival, but we did not observe this, possibly owing to differences in patient population/sample size and/or evolution of care practices.

There are currently no established treatment protocols for TM, and the guidelines for both surgical intervention and systemic treatment remain undefined.^11,20,21^ The mainstay of treatment currently is surgical, and 94% of patients in our cohort underwent radical orchiectomy, but only 33% had RPLND, 25% had PLND, and 22% had ILND. Because this diagnosis is often made incidentally following scrotal surgery, revision surgery and re-resection is often necessary. Fifty percent of patients in our cohort with nonmetastatic disease at diagnosis eventually developed metastatic disease despite undergoing upfront orchiectomy. This proportion might be even higher, accounting for patients who were lost to follow-up. Median RFS was 2.2 years after radical orchiectomy. These findings highlight the need to develop evidence-based treatment guidelines beyond orchiectomy to identify which patients would benefit from lymphadenectomy and adjuvant systemic or radiation treatment. The inguinal/retroperitoneal lymph nodes are the most common sites of metastatic disease at diagnosis or recurrence; thus, guidelines for retroperitoneal lymph node biopsies and/or PLND or ILND are needed for accurate staging and disease management. Surgery is both a diagnostic and potentially therapeutic procedure in this disease process, but the risk/benefit considerations of aggressive surgical management in this elderly population relative to younger patients with testicular germ cell tumors or other genitourinary malignancies is a complex consideration.

Testicular mesothelioma-specific guidelines for systemic treatments are lacking and largely extrapolated from the more common and more studied DPM and MPeM.^6,19^ In our cohort, nearly 90% of patients were diagnosed after the approval of pemetrexed in 2004, yet only 42% received any systemic therapy. Notably, only one patient received treatment in the adjuvant setting, whereas the remainder received systemic treatment in the metastatic or recurrent setting. Most patients who did not receive systemic treatment either lacked a history of metastatic or recurrent disease or were lost to follow-up. Among patients who received systemic therapy, 60% underwent only one line of treatment. The most common front line systemic regimens in our cohort were platinum-pemetrexed doublet chemotherapy and immune checkpoint inhibitors, both of which are standard, approved first-line therapies for DPM. There is an unmet need to identify effective systemic treatments for patients with metastatic TM based on histology and molecular alterations. Larger cohorts are needed to study the response to different systemic therapies in TM, including by histologic subtype.

Given the rarity of TM and its nonspecific clinical presentation, accurate pathologic diagnosis, and consultation with a multidisciplinary team including an oncologic urologist, medical oncologist, radiologist, and pathologist at a large center with experience in this rare disease are critical for devising appropriate treatment and monitoring plans. These patients should receive expert consultation and should be considered for multimodality therapy rather than standard orchiectomy alone.

Data on the genomic landscape of TM is scarce. Previous small subset studies reported that somatic alterations are most commonly detected in *NF2*, *CDKN2A/B*, and *BAP1*, consistent with our findings.^22,23^ We report a higher frequency of *CDKN2A/B* alterations in patients with TM compared with patients with MPeM and a lower frequency of *BAP1* alterations compared with patients with DPM and MPeM.^1,13^ Although data on *MTAP* alterations were unavailable for most patients in the NGS panel at the time of the study, it is likely that many patients with *CDKN2A* deletions have concurrent *MTAP* loss,^24^ highlighting the importance for broad based modern NGS assays for patients with TM to determine potential trial applicability. While the numbers are small, these results suggest that TM may be genomically distinct from DPM and MPeM and, therefore, may present unique options for targeted therapies and systemic therapies relative to other mesotheliomas. More widespread tumor molecular testing is needed to better define the molecular landscape of TM as are trials that allow for the inclusion of this rare disease.

Germline mutations in *BAP1* and other genes involved in DNA damage repair and tumor suppressor pathways are associated with an increased risk of DPM and MPeM,^25^ but it is not known whether alterations in these genes or others also predispose individuals to TM. Germline NGS was performed on six patients in our cohort, revealing a pathogenic germline alteration in *NTHL1* in one patient. This gene is involved in DNA damage repair, and biallelic gene mutations are most associated with increased risks of colon and breast cancers.^26^ Studies of larger sample sizes are needed to identify germline gene alterations potentially associated with an increased risk of TM.

Our study has some limitations, including the small sample size of our cohort, which was particularly restrictive for subset analyses. The study is also limited by its retrospective observational design. Our cohort was drawn from a large comprehensive cancer center; thus, some findings may not be applicable to a broader population. Some patients had limited long-term clinical data, because they presented for consultation and then continued follow-up with a physician from a different organization. Moreover, tumor and germline NGS data were unavailable for most patients.

This study represents the largest known single-institution series and largest genomic dataset reported for patients with TM to date. Our findings show the unique clinicopathologic and genomic features of this disease, including a younger age at diagnosis and lower frequency of *BAP1* alterations compared with DPM. We also show low rates of RPLND and systemic treatments despite high rates of metastatic disease. Testicular mesothelioma has features suggestive of a distinct disease entity that warrant further investigation, and treatment guidelines should not simply be extrapolated from DPM. We propose the creation of a patient registry to aggregate these cases. Prospective multicenter studies, clinical trials, and more widespread tumor and germline molecular testing are needed to improve the understanding of TM, establish disease-specific treatment protocols, and improve outcomes for patients.

## Supplementary Information

Below is the link to the electronic supplementary material.

Supplementary file 1 (DOCX 1319 kb)
