# Supplementary material for: Clinicopathologic Characteristics and Clinical Outcomes of Patients with Testicular Mesothelioma
Source: Ann Surg Oncol. 2025 Jul 30;32(13):10285–93. doi: 10.1245/s10434-025-17978-3 (PMC12589385; doi:10.1245/s10434-025-17978-3)
Supplement: Supplementary file 1 — (DOCX 1319 kb) [file 10434_2025_17978_MOESM1_ESM.docx]

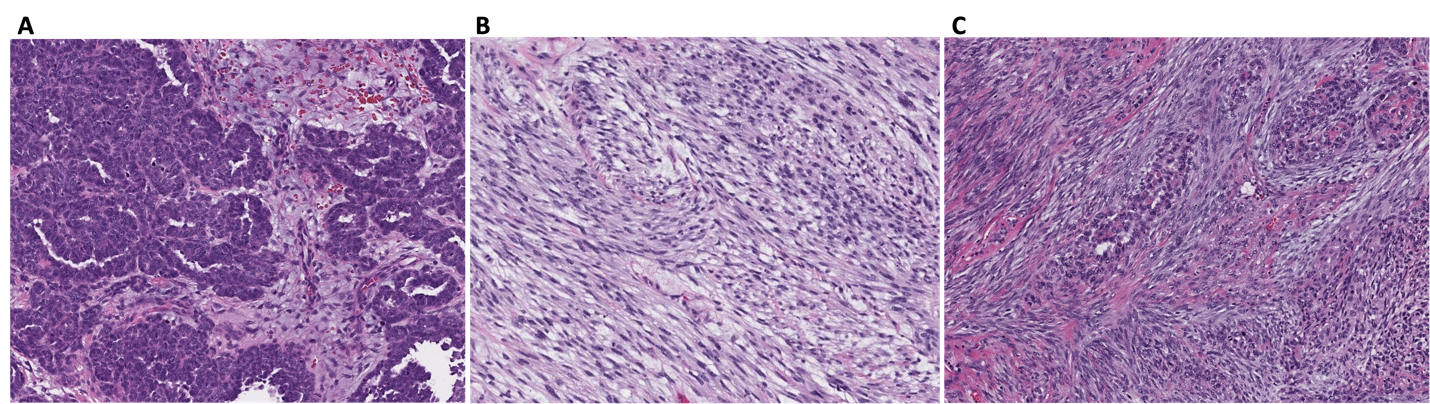


Supplementary Figure 1. Hematoxylin and eosin-stained images of A) Epithelioid subtype of testicular mesothelioma with tumor cells arranged in tubular and solid architecture (100x), B) Sarcomatoid subtype of testicular mesothelioma in the form of spindle cells arranged in fascicles (100x), and C) Biphasic subtype of testicular mesothelioma comprising both epithelioid tumor cells admixed with spindle cells/sarcomatoid components (100x).
